# Comprehensive analysis and recommendation of feature evaluation measures for intrusion detection

**DOI:** 10.1016/j.heliyon.2020.e04262

**Published:** 2020-07-09

**Authors:** Adel Binbusayyis, Thavavel Vaiyapuri

**Affiliations:** College of Computer Science and Engineering, Prince Sattam bin Abdulaziz University, AlKharj, Saudi Arabia

**Keywords:** Computer science, Cybersecurity, Intrusion detection, Deep belief network, Feature selection, Distance, Correlation, Consistency, Information gain, Detection engine, Response engine

## Abstract

The revolutionary advances in network technologies have spearheaded the design of advanced cyberattacks to surpass traditional security defense with dreadful consequences. Recently, Intrusion Detection System (IDS) is considered as a pivotal element in network security infrastructures to achieve solid line of protection against cyberattacks. The prime challenges presented to IDS are curse of high dimensionality and class imbalance that tends to increase the detection time and degrade the efficiency of IDS. As a result, feature selection plays an important role in enabling to identify the most significant features for intrusion detection. Although, several feature evaluation measures are being proposed for feature selection in literature, there is no consensus on which measures are best for intrusion detection. Therein, this work aims at recommending the most appropriate feature evaluation measure for building an efficient IDS. In this direction, four filter-based feature evaluation measures that stem from different theories such as Consistency, Correlation, Information and Distance are investigated for their potential implications in enhancing the detection ability of IDS model for different classes of attacks. Along with this, the influence of the selected features on classification accuracy of an IDS model is analyzed using four different categories of classifiers namely, K-nearest neighbors (KNN), Random Forest (RF), Support Vector Machine (SVM) and Deep Belief Network (DBN). Finally, a two-step statistical significance test is conducted on the experimental results to determine which feature evaluation measure contributes statistically significant difference in IDS performance. All the experimental comparisons are performed on two benchmark intrusion detection datasets, NSL-KDD and UNSW-NB15. In these experiments, consistency measure has best influenced the IDS model in improving the detection ability with regard to detection rate (DR), false alarm rate (FAR), kappa statistics (KS) and identifying the most significant features for intrusion detection. Also, from the analysis results, it is revealed that RF is the ideal classifier to be used in conjunction with any of these four feature evaluation measures to achieve better detection accuracy than others. From the statistical results, we recommend the use of consistency measure for designing an efficient IDS in terms of DR and FAR.

## 1 Introduction

With the revolutionary advance in network infrastructure and information technologies, the cybersecurity threats are also consistently increasing in number and intricacy [1]. For instances, in 2018, the MCAFee's report on economic impact of cybercrime states that malicious activities are quite astounding with 80 billion malicious scans each day [2]. Also, the 2018 Cybersecurity breaches survey states that 43% of high profile businesses across the world have fallen victim to cybersecurity breaches in the last 12 months [3]. Furthermore, according to annual cybercrime report of 2017 its estimated that the financial losses by cybercrime activities will cost $6 trillion per year by 2021 [4]. This high cost has necessitated an urgency need for developing new cyberattack defense methods and techniques [5], [6].

Although several antivirus software, firewalls and IDS exist to detect and protect IT infrastructures from many known kind of cyberattacks, cybercriminals in turn have become more skilled in developing new advanced and more complex techniques to gain access and damage critical IT infrastructure [7]. Recent annual Cisco security of 2018 has point out that application of machine learning will pave a way to develop cyberdefense methods that can automatically detect any unusual new patterns in network traffics [8]. In this line of direction, recently hot research topics are to develop a new effective and adaptive defense methods than ever before [9]. Traditionally, Cyber defense products such as firewalls were considered as the first line of security defense against cyberattacks in most business networks but fail to identify the attacks on allowed services. Under such situations, second line of security defense is mandate by products like anti-virus and IDS [10]. But, anti-viruses are delimited to protect network only from those malwares whose signatures are available in the database. Also, the update schedule for signatures is either on daily or weekly basis [11]. Therefore, the network is unsafe against malicious activities in the time between the updates. Hence, IDS are considered as a key asset to protect IT infrastructure against threats and enhance network security in almost all organizations.

In this line, many researches are being carried out to develop intelligent IDS and achieve better network security [12]. For instances, Yang Jia et al. attempted to build an intelligent IDS applying deep neural network and succeeded with promising results [13]. Similarly, a new intelligent IDS was presented in [14] applying ensemble and unsupervised machine learning techniques specifically to combat the security challenges in software-defined 5G networks. Saurabh Dey et al. in [15] proposed a multi-layered IDS for mobile clouds involving heterogeneous client networks. In this approach, they have applied machine learning methods such as DBScan and K-means to observe the incoming traffic pattern and detect potential attacks. Also they have indicated that the complexity of this approach can be customized according to the requirement of the client network. Also in [16], the authors have attempted to design an IDS combining learning, case-based reasoning and reactive behavior for acquiring knowledge from past solutions and support the evolution of case-based reasoning to reactive behavior in enhancing the performance of the IDS. In [17], Vajiheh Hajisalem et al. investigated to develop intelligent CART classifier for IDS by optimizing rapidity and accuracy. To achieve this, they have combined artificial fish swarm and artificial bee colony to choose effective If-then rules for the classifier CART and achieved a DR of 99% with FAR of 0.01%. Approach proposed in [18] successfully applied machine learning to design an intelligent IDS that are capable to learn and update incrementally the detection engine and maintain good detection rate with low FAR over long time period.

While numerous work has been devoted in the past decade on devising IDS applying machine learning techniques [1], the success of these methods depends on the quality of data used. Unfortunately, the current real-world network traffic is characterized by huge volume of high dimensionality data. This may negatively impact on the detection accuracy of IDS due to the presence of irrelevant/redundant information in network traffic. Also, it may slow down the entire detection process due to high computational complexity required to handle such data. Therefore, it is of paramount importance in the intrusion detection process to identify or propose an effective method to handle the reduction of data dimensionality as recognized by great body of scientific literature.

Further, the high dimensionality is not the only challenge presented to IDS. Another important issue that may worsen the detection accuracy of IDS, but is often ignored in this domain, is imbalance in class distribution [19], [20], [21]. This occurs when the data contain different numbers of observations for the different classes which is a common situation in intrusion detection process as the attack traffic tends to be only a small portion of overall traffic. The class with dominate number of observations than other classes is called majority class, while the class with smallest amount is called minority class. The class imbalance causes the classifier to bias towards majority class and tends IDS to generate many false alarms. In this situation, sophisticated attackers are encouraged to create minority attack types to reach their targets. Despite, misclassification of minority attack types leads to severe loss in practical applications, the class imbalance problem has not received substantial attention as it would deserve in the field of IDS.

The solutions crafted to combat the class imbalance problem, fall under two main categories namely, data level and algorithmic level [22]. The data level focuses on changing the original data distribution and includes many different resampling techniques. Whereas algorithmic level adjusts the existing learning algorithm to strengthen their ability and optimize their classification accuracy towards minority class. Recently, the importance of feature selection for class imbalance problem is realized and has received much attention in the field of machine learning [19], [22]. In general, feature selection aims to improve the quality of the dataset by selecting the most informative features and eliminating the features that are irrelevant or redundant. With an imbalanced dataset, the key idea of employing feature selection is to find the optimal subset of features that can optimize the contrast of minority class from other classes in the data and reduces the risk of misclassifying minority class samples. Hence, this notion forms the initial impetus for this research work.

To date, very few studies are reported in literature to illustrate the significance of feature selection in handling high dimensional class imbalance problem. Further, most of these studies are conducted on biological data. Therefore, we believe it would be of unique contribution if the significance of feature selection for high dimensional imbalanced intrusion dataset is investigated.

Accordingly, the objective of this work will be to give guidance for researchers not working in feature selection field but searching out for the best feature evaluation measure to build efficient IDS. In this direction, the work here investigates the performance of different feature evaluation measures based on their correlation with intrusion detection accuracy and recommends the most appropriate measure to consider for use while building an IDS. Here, KNN, RF, SVM and DBN was used as a classifier to demonstrate the quality of the recommending feature evaluation measure. The standard intrusion detection datasets NSL-KDD and UNSW-NB15 which are high dimensional and imbalanced was employed to prove the recommending feature evaluation measure in terms of higher DR and lower FAR. Finally, statistical analysis was conducted to confirm the recommending feature evaluation measure for building efficient IDS.

## 2 Intrusion detection system

According to NIST [23], “Intrusion is defined as an attempt to compromise confidentiality, integrity and availability (CIA), or to bypass the security mechanisms of a computer or network”. “Intrusion detection is a process of monitoring the events occurring in a computer system or network and analyzing them for signs of intrusions”. Therefore, IDS is a security management system for monitoring anomalous activities that take place within computers or network systems and flag out the activity that comprise the computer security principles of CIA. They are potential in detecting malicious activities from both outsiders and insiders of the network system.

**Figure 1:**
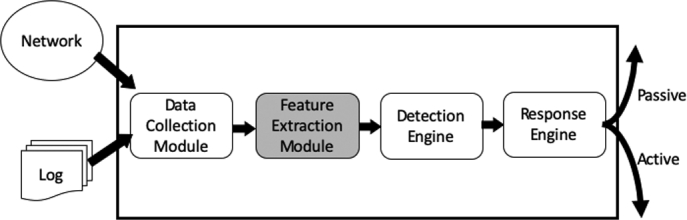
General IDS architecture.

IDS consist of four major components namely, Information Source, Feature Selection, Detection Engine and Response. These four components function collaboratively with an objective to identify attacks and report output in a required format [24]. Fig. 1 shows the organization of these components in IDS.

(A) **Data Collection:** It is responsible for collecting intrusion evidence data from desired sources and provide the collected data in comprehensive format to the rest of the system. Collecting all information is expensive, and the key challenge is in collecting the distinguished information.
(B) **Feature Extraction:** It is responsible to selectively retain the informative set of features for the purpose of attack characterization eliminating the irrelevant/redundant features. Finally, it forms the feature vector with selected subset of features. This work will focus to contribute a new approach for feature extraction with an aim to enhance the detection performance of the system.
(C) **Detection Engine:** It is the core component of IDS and its responsible for analyzing the data to detect intrusion activity. The strength of the overall IDS is often determined by the capability of this component to detect all classes of attacks.
(D) **Response Engine:** It is responsible to decide how to respond when the detection engine identifies an attack and controls the reaction mechanism. This component decides either to take “passive response” by just triggering an alert without taking any action against the source or to take “active response” by blocking the source for a predefined time period. The type of response action to be taken is based on the security policy decided by the organization.

IDS are usually categorized according to their deployment in real time and detection mechanism. With regard to their deployment, they are classified as Host-based and Network-based IDS [25]. The host-based IDS are deployed in the local machine to collect information about the host machine activities and detect any abnormal activities in that machine. Alternatively, the network-based IDS monitors network traffic and analyzes all packets in the network to identify any threats on network resources. Further, these IDS are two types based on detection mechanism namely, signature-based and anomaly-based. The signature-based IDS employs pattern matching techniques to detect anomaly activity by comparing the activities across network system with predefined attack signatures stored in IDS database. The key benefits of these methods are their simplicity and low false alarm rates (FAR). But their application in real time is confronted by their inability to recognize and block new or unknown attacks whose signatures are not available in IDS database. Alternatively anomaly-based IDS monitors all activities across the network system and employs statistical learning techniques to pinpoint any action that deviates significantly from normal activities. One of key benefits of anomaly-based IDS is their ability to recognize new and unknown attacks. Therein they are preferred for real-time applications. Unfortunately, these systems exhibit high FAR due to their inability to define a clear boundary between normal and abnormal behavior. Researchers attempted to combat this issue by applying machine learning techniques to improve the performance of detection engine in IDS. Several machine learning techniques are proposed in literature to design detection engine, a detail survey of all techniques with their pros and cons is given in [1], [26], [27]. From these cited literatures, it is clear that less attempts are made to enhance the performance of detection engine providing the most important and relevant features required for intrusion detection. This indicates that there seems to be a gap in this area. The current work attempts to resolve this gap recommending an appropriate feature evaluation measure to remove redundant/irrelevant information and enhance the accuracy of detection engine with reduced false alarm rate.

## 3 Feature selection techniques

**Figure 2:**
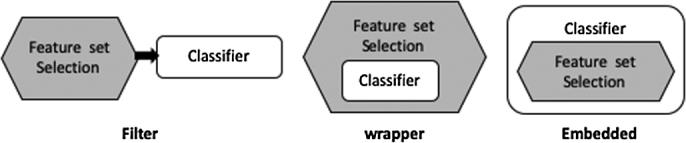
Strategies for feature selection.

Selection of important features is a first and important data preprocessing step in IDS development process. The key objective of feature selection techniques is to select compact and optimal subset of relevant features from the given large dataset and enhance the accuracy of the intrusion detection classifier. A standard feature selection technique comprises two key parts [28]. One is selection algorithm to describe how subset of features is selected for consideration. Another is feature evaluation measure to describe how the selected feature subsets are evaluated for quality. In general, feature set selection algorithms as shown in Fig. 2 are divided into three types based on different selection strategies: filter, wrapper and embedded methods. The wrapper methods search through feature space to select a subset that gives the highest detection classifier accuracy. Embedded methods utilize the structure of specific classes of detection classifiers to guide the feature selection process and select a feature subset during the learning stage. One critical problem with these two categories of methods is application of exhaustive search strategy to select the optimal subset among all the possible feature subsets which results in high computational complexity. Also, these methods provide features that are classifier dependent and may suffer from the risk of overfitting. Alternatively, filter methods select features based on predefined metrics rather than using classifiers. Therefore, the selected features are more general and have no dependence with classifier used for detection. Importantly, they are less expensive methods and are therefore most preferable for large datasets. Due to these advantages, this work utilizes filter methods.

## 4 Feature evaluation measures

**Figure 3:**
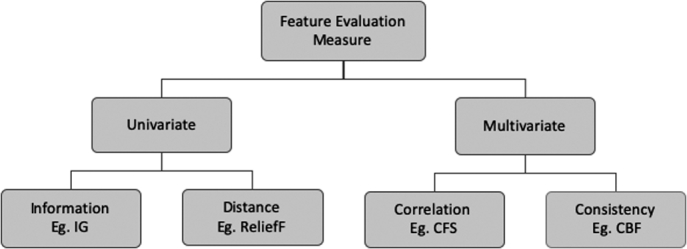
Taxonomy of filter evaluation measure.

Evaluation measure plays a crucial role in feature selection techniques for guiding the search in feature space and for selecting the discriminative features. The filter methods evaluate the merits of features or feature subsets using various measures based on the intrinsic characteristics of data rather than considering the interaction of data with the classifier. Thus far, several robust filter-based feature evaluation measures have been proposed to remove irrelevant and redundant features. As shown in Fig. 3, they can be grouped into two categories based on what combination of feature and class information is used to compute the measure. Univariate measures assess the discriminative ability of each feature individually and assign a weight to each feature. This weight is not influenced by other features in the set. Multivariate measures assess the discriminative ability of the entire set of selected features. The most widely used four filter-based feature evaluation measures are adopted in the present work for investigation and they are described in the following subsections.

### 4.1 Consistency measure

Consistency measure is a multivariate filter measure that evaluates the merits of a candidate feature subset by computing its inconsistency rate over the given dataset as given in Eq. (1). It uses heuristic search technique to guide them through the given feature space and find the correct candidate feature subset. For example, it starts with the original number of features in the given dataset and continues to generate random subset with C features until a minimum size feature subset satisfying the inconsistency criterion is reached. Thus, the inconsistency criterion that checks the inconsistency rate of candidate feature subset against the user defined rate (InConsistCheck(S,D)<γ) is the key success of consistency measure. In other words, this criterion defines the extent to which dimensionality reduction is acceptable. Based on this criterion, the outcome of consistency measure is a minimum size feature subset that can separate the target classes as consistently as full feature set. In literature, it has proven to be the best and fast filter in removing irrelevant and redundant features even in presence of noise in the dataset. The feature selection algorithm based on consistency measure (CBF) devised by Liu et al. [29] is given in Algorithm 1. The time complexity of this algorithm is O(NI ⋅ $M^{2}$); where M is the number of selected features and NI represents the total number of instances in the dataset.

(1) $$ConsistencyRate_{s}=\frac{\sum_{i=0}^{J}|Z_{i}|-|P_{i}|}{NI}$$

where s represents the candidate feature subset and J is the number of distinct combinations of feature values for $S_{i}$. $\left|Z_{i}\right|$ and $\left|P_{i}\right|$ denotes the number of occurrence and the cardinality of the majority class for the ***i***th feature value in the combination.

**Algorithm 1:**
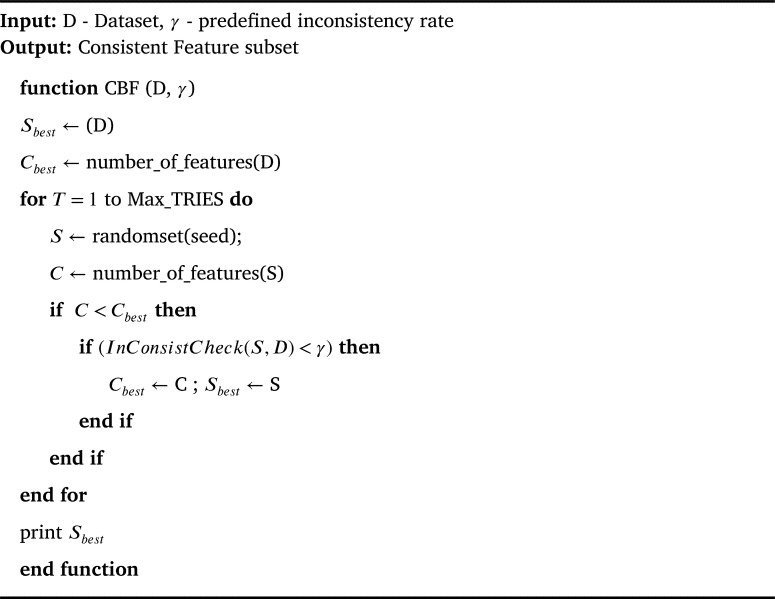
CBF Algorithm [29].

### 4.2 Correlation measure

Correlation Measure is a multivariate filter measure that evaluates the merits of the candidate feature subset based on the degree to which each feature in subset is correlated with the target class and is uncorrelated with other features. Thus, this measure is very powerful in removing redundant and irrelevant features on the ground truth that irrelevant features will have weak association with target class and redundant features will be strongly correlated with at least one of other features. Correlation measure by default uses search techniques to explore the feature space and heuristic evaluation function defined in Eq. (2) to assess the merit of the candidate feature subset. For example, it starts with an empty set of features and continues to explore the feature space for all possible single feature expansions until no further improvements can be achieved in merit evaluation.

(2) $$Merit_{s}=\frac{k\bar{r_{cf}}}{\sqrt{k+k(k-1)\bar{r_{ff}}}}$$

where $Merit_{s}$ is the evaluation of a feature subset with k features, $\bar{r_{cf}}$ is the average correlation value between features and class labels, and $\bar{r_{ff}}$ is the average correlation value between two features. Hall et al. [30] devised a feature selection algorithm given in Algorithm 2 using correlation measure (CFS) with time complexity of O(NI ⋅ $M^{2}$).

**Algorithm 2:**
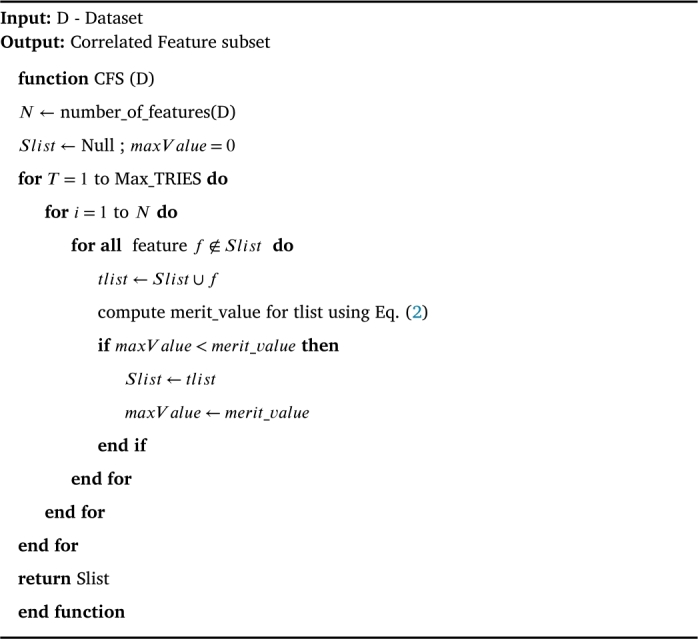
CFS Algorithm [30].

### 4.3 Information measure

Information Measure is a univariate filter measure that evaluates each individual feature for the quantity of information it shares to detect the target attack class. As opposed to other measures, they are capable of quantifying the amount of information making no assumption about the data distribution and size. Also, they are capable of discovering any relationship between classes and a feature no matter it is linear or non-linear. For this purpose, the information measure calculates the information gain (IG) or mutual information between classes C and feature F using the equation given below to determine the relevance of a feature in class C [31], [32].

(3) IG=H(C)−H(C|F),=H(F)−H(F|C),=H(F)+H(C)−H(F,C)

Here, H(C) is the entropy of class C and is calculated as,

(4) $$H(C)=-\sum_{c\in C}p(c)\;log_{2}\;p(c)$$

H(C|F) is the conditional entropy of class C given the feature F and is calculated as,

(5) $$H(C|F)=-\sum_{f\in F}p(f)\sum_{c\in C}p(c|f)\;log_{2}\;p(c|f)$$

Higher the value of IG, higher is the relevance of the feature F to detect the target class C. The pseudocode for computing IG is given in Algorithm 3. This algorithm has a time complexity of O(N ⋅ T2), where N is the number of features in the dataset and T2 is the time taken to calculate the IG.

**Algorithm 3:**
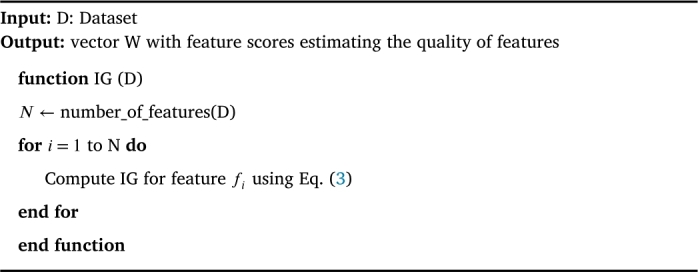
IG Algorithm [33].

### 4.4 Distance measure

Distance Measure (DM) is the only univariate filter measure that has the capability of learning the feature dependencies in the process of identifying the ‘quality’ feature for detecting the target attack class [34]. Most importantly, It does not make any conditional independence assumption upon target attack classes rather they are efficient in using the contextual information to correctly estimate the quality of a feature. Accordingly, it computes statistic score value for each feature by rewarding a feature if it has different values for two near instances from different classes and by penalizing an feature if it has different values for two near instances from the same classes. The steps for finding the statistic score value for each feature using distance measure (ReliefF) are given in Algorithm 4. It comprises three key steps. First, it randomly selects an instance from the training dataset. Second, it finds two nearest neighbors, one from same class called nearest Hit (H) and one from different class called nearest miss (M). Third, it updates the statistic score for all features based on their values for M and H using the equation given below [35],

(6) $$W_{f}=W_{f}-(\frac{diff_{f}(R,H)}{m}-\frac{diff_{f}(R,M)}{m})$$

where m is the number of random training instances used to update W and diff is the difference between two instances as defined below and is normalized to the range [0,1].

(7) $$diff_{f}(I1,I2)=\frac{\left|value(f,I1)-value(f,I2)\right|}{max(f)-min(f)}$$

**Algorithm 4:**
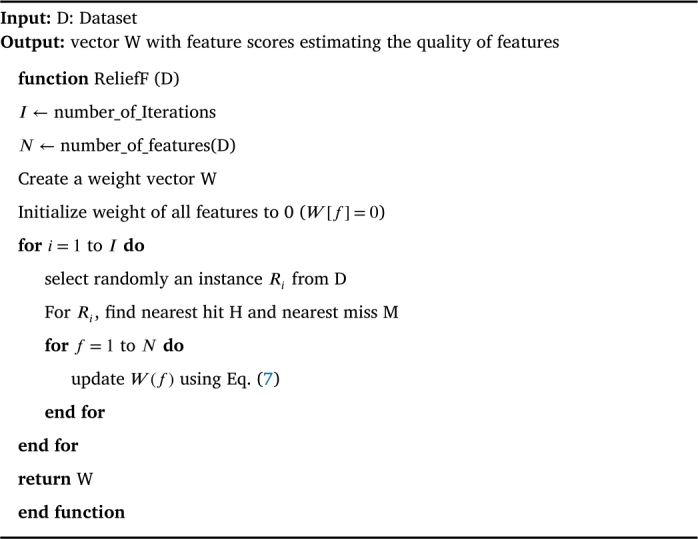
ReliefF Algorithm [35].

Now, the weight $W_{f}$ of a feature increases if it has same values for instances from same class and distinguishes the instances from different classes. The above three steps are repeated by selecting randomly m instances from training set. As this measure uses the concept of nearest neighbors rather than search algorithm techniques. The time complexity is based on the number of sampled instances and is given by O $\left(m^{2}.N)\right)$.

## 5 The classifier scheme for intrusion detection

Since there is no one best classification method that fits all applications, it is recommended to examine multiple classifiers considering their characteristics, complexity, performance and previous applications in literature. Accordingly, this work chose three classifiers from different categories such as statistical learning theory (SVM), distance (KNN), ensemble learning (RF) and deep learning (DBN) to investigate the effectiveness of different feature evaluation measures for classification performance in IDS. Following is a brief description on these classifiers.

### 5.1 k-Nearest neighbor (KNN)

KNN is a simple and easy-to-implement classification scheme [36]. Unlike other classification schemes, KNN is a lazy learner which means it does not require explicit training phase to learn a discriminative function rather it memorizes the training samples. KNN use nearest neighbor decision rule and the majority vote of k-nearest neighbors to classify any new unknown intrusion. More importantly, they are non-parametric, meaning it does not make any assumption on the underlying data pattern distribution rather it determines the model structure from the data. These were the reasons for choosing KNN in this work for evaluation.

### 5.2 Random forests (RF)

RF is an ensemble-based learning method [37]. It operates by constructing and combining several randomized decision trees. Aside from being simple to use, RF is a versatile method in producing accurate results for many types of data. RF has turned out to be very powerful model with their ability to limit the notorious overfitting without substantially increasing error due to bias. Further, it is worth noted that RF is capable of handling data imbalances in different classes especially for large datasets [6]. RF has found a wide spread acceptability in various applications due to its robustness to noise, tuning simplicity, parallel architecture and due to its ability to efficiently handle non-linear classification tasks. Attributed to these advantages of RF, this work has also chosen RF for comparison.

### 5.3 Support vector machine (SVM)

SVM is a discriminative classifier [38] that blends linear modeling with instance-based learning to find an optimal separating hyperplane (OSH) with maximal margin between classes in feature space. The data points that are closest to the OSH are called support vectors and are used to create decision boundary. The OSH is oriented at the maximum distance between the sets of support vectors. It is because of this orientation, SVM generalizes more accurately for new unknown cases even when with limited number of training samples. Also, SVM is acknowledged for producing significant accuracy with less computation power. Another key property of SVM is their ability to use kernel function to automatically map the data samples to higher dimensional space and solve non-linear problems in that space where the classes can be separated linearly. Due to these inherent properties, SVM is preferred as the most reliable and accurate algorithm in most applications. Hence, SVM is also chosen in this work for comparative evaluation. But since SVM is initially defined for binary classification, the present work employs kernel function and constructs SVM classifier with “one-to-one” combination to realize the multi-classification in intrusion detection.

### 5.4 Deep belief network (DBN)

DBN is a deep learning mechanism with potential to determine optimal representation for input data than the shallow models [39], [40]. It is a probabilistic generative network created with multiple layers of Restricted Boltzmann Machine (RBM) for learning complex data pattern. Here the layers are trained sequentially in greedy fashion. Training process in DBN consists of two stages. The first stage called pre-training employs unsupervised learning to train each RBM one by one with large amount of data without labels to capture the data distribution and obtain their initial weights. Later, the second stage called fine-tuning employs supervised learning with data labels to adjust the initial weights through error backpropagation and finalize their weights for enhanced discriminative ability. Thus, the pre-training stage of DBN not only reduces the training complexity but also enhances its discriminative ability by avoiding overfitting.

Recently, DBN is most valued for its versatile ability and has exposed great success in unsupervised feature dimensionality reduction and supervised pattern classification [41], [42]. But only limited studies in literature have used DBN in the field of intrusion detection [43], [44]. Also, the authors in these studies have not focused to investigate the influence of feature selection on DBN. Therefore, we believe if DBN is chosen for comparative evaluation then the experimental results from this study can contribute to deep learning research communities.

## 6 Experimental setup

This section describes the datasets, performance metrics, framework design adopted for the experiments that were conducted to investigate the effectiveness of the four feature evaluation measures for intrusion detection.

### 6.1 Datasets

Most of the real-world network traffic data is unavailable due to the companies' privacy and security issues. On the other direction, there are number of public datasets available for IDS performance evaluation. But, these datasets suffer from lack of sufficient number of traffic types and modern low footprint attack styles. Therefore, in order to facilitate a fair and reasonable comparison, this article uses an older benchmark dataset, NSL-KDD and a new contemporary dataset UNSW-NB15 to compare the effective performance of the four feature evaluation measures under study. A brief description of these two high dimensional imbalanced cybersecurity datasets is given below.

#### 6.1.1 NSL-KDD dataset

KDD-Cup'99 [58] is one of the most widely accepted benchmark dataset in the field of intrusion detection despite of being outdated and inherent with several problems [45]. In 2009, the Network Security Lab—Knowledge Discovery and Data Mining (NSL-KDD) released an improved version of KDD-Cup'99 dataset known as “NSL-KDD dataset” [46]. This presented NSL-KDD dataset mitigates the inherent problems in KDD-Cup'99 such as large number of redundant records and unbalanced distribution of records, which might otherwise mislead the evaluation. Thus, NSL-KDD is considered as most valuable and reliable benchmark resource for performance evaluation in many studies related to intrusion detection and other cybersecurity related tasks. Thus, after removing the duplicate records, the NSL-KDD dataset comprises 125,973 records of training and 22544 records of testing, each record with 41 features and a class label, determines whether the traffic is normal or an attack type. It includes 22 different types of attacks belonging to one of the four major classes of attack in what follows

(a) *Denial of Service (Dos):* attacker make the resources too busy to process the request from legitimate users to the resources.
(b) *Probe:* attacker attempts to gather important information about the network and discovers vulnerabilities to launch an attack
(c) *User to Root (U2R):* attacker exploits the vulnerability in the system to gains the super user privileges
(d) *Remote to Local (R2L):* attacker exploits the vulnerability in the system to gain local access as a user to a remote computer.

The distribution of records of these five attack class types is shown in Table 1. From this statistics, it can be observed that the prevalence of DoS class is around 36% but the attack classes such as R2L and U2R accounts for less than 1%. This clearly shows that this dataset is extremely unbalanced.

**Table 1:** Distribution of four attack classes in NSL-KDD.

Attack class	Number	Volume (%)
**DoS**	45927	35.45
**Prob**	11656	9.25
**R2L**	995	0.75
**U2R**	52	0.05

#### 6.1.2 UNSW-NB15 dataset

UNSW-NB15 is a comprehensive latest published dataset for research purpose by Australian Centre for Cyber Security (ACCS) to reflect a more complex and modern threat environment [47]. This dataset contains a hybrid of realistic modern legitimate activities and contemporary synthesized attack behaviors of live network traffic. The UNSW-NB15 dataset is an extensive collection of 48 features extracted from network packet headers and network payload to effectively reflect network traffic record and a class label to classify the traffic record either as legitimate or attack. The dataset involves nine modern classes of attacks as defined below,

(a) *Fuzzers:* attacker attempts to find out the security loopholes in operating system, network or programs and crash it by feeding massive amount of random data.
(b) *Analysis:* attacker attempts to gain access into web applications via emails (e.g., spam), ports (e.g., port scans), and web scripts (e.g., HTML files).
(c) *Backdoor:* a technique adopted by attacker to bypass a stealthy normal authentication procedure and gain unauthorized remote access to a host or network.
(d) *DoS:* attacker make the resources too busy to process the request from legitimate users to the resources.
(e) *Exploit:* attacker takes the advantage of security vulnerability caused by an unsuspected or intentional behavior on a host or network.
(f) *Generic:* a technique employed by attacker to cause collision using hash function against every block-cipher irrespective of block-cipher configuration.
(g) *Reconnaissance:* attacker attempts to gather important information about the network and discovers vulnerabilities to launch an attack.
(h) *Shellcode:* attacker injects piece of code to start a command shell and exploit the compromised machine.
(i) *Worm:* attacker attempts to replicate itself and spread on other computers based on the security failures on the host.

The UNSW-NB15 dataset is available in two forms, original and partitioned UNSW-NB15 datasets. The original UNSW-NB15 dataset contains 2,540,044 records logged in four csv files. The partitioned UNSW-NB15 dataset is mainly configured for research purpose with 175,341 of training and 82,332 of testing records, in which each record is characterized by only 42 features and a class label. The network traffic distribution of this dataset under nine attack class types is shown in Table 2. According to this statistics, the frequency of normal traffic records accounts for 32%. On other hand, the frequency of attack traffic accounts very less percentage and varies greatly. For example, the number of attack samples of Exploits and Worms differ by about 257 times. Thus, this dataset is also exhibits high imbalance.

**Table 2:** Distribution of nine attack classes in UNSW-NB15.

Attack class	Number	Volume (%)
**Fuzzers**	18184	10.37
**Analysis**	2000	1.14
**Backdoors**	1746	0.99
**DoS**	12264	6.99
**Exploits**	33393	19.04
**Generic**	40000	22.81
**Reconnaissance**	10491	5.98
**Shellcode**	1133	0.64
**Worms**	130	0.07

### 6.2 Evaluation metrics

Several experiments were conducted to investigate and compare the effectiveness of the four feature evaluation measures for intrusion detection. For this purpose, the most widely used three metrics namely, the accuracy, detection rate, false positive rate were adopted as in most previous literature on IDS. These metrics are defined as follows,

• **Accuracy (ACC):** is measured as the proportion of connection records that are correctly classified as given below,
  (8) $$ACC=\frac{TP+TN}{TP+TN+FP+FN}$$
• **Detection rate (DR):** Also called True Positive Rate is measured as the proportion of network attack records that are correctly classified as given below,
  (9) $$DR=\frac{TP}{TP+FN}$$
• **False positive rate:** also termed as false alarm rate (FAR), it is measured as probability of incorrectly classifying normal network connection records as attack. The consistent increase of this metric may mislead the network administrator to intentionally ignore the alerts from network system. As a result, the entire network may be face dangerous situation. Therefore, it is always advisable to keep this metric value as low as possible.
  (10) $$FAR=\frac{FP}{FP+TN}$$

Another most important metric required called Kappa statistics (KS) [48] is employed as one of the evaluation measure as it is more essential than precision and recall to furnish the comprehensive performance of the model with unbalanced- and multi-class problem. Since the two benchmark datasets utilized here are unbalanced, KS is considered here as an essential measure for comparison. In essence, KS is measured as agreement between predicted class of a dataset and the observed label as ground truth, while correcting the agreement that occurs by chance as given below,

(11) $$KS=\frac{P_{O}-P_{E}}{1-P_{E}}$$

where $P_{O}$ is the proportion of observed agreements and $P_{E}$ is the proportion of agreements expected by chance.

### 6.3 Framework

The experiments designed for evaluation process consist of three main steps: First, the chosen dataset is preprocessed by mapping the symbolic feature to numeric value, discretizing continuous feature and normalizing each feature to specific range [0,1]. Next, feature selection was performed using the four competing feature evaluation measures discussed in the Section 4 to select the most informative features from the preprocessed dataset. As recommended in literature [49], five-fold cross validation strategy was applied five times for feature selection to avoid selection bias. Finally, the feature subsets resulting from four feature evaluation measures were evaluated with the above discussed four different classifiers namely KNN, RF, SVM and DBN.

**Figure 4:**
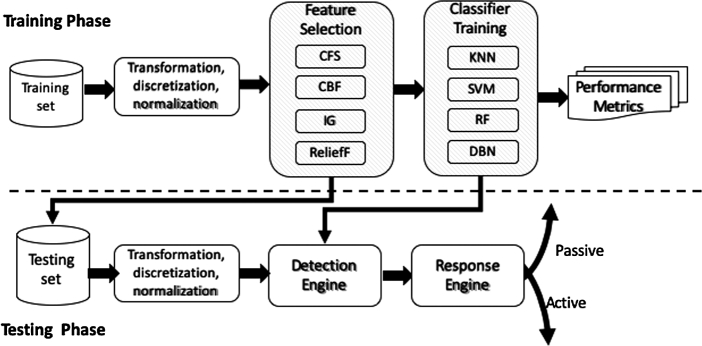
Experimental framework used for evaluating the performance of four feature evaluation measures.

As evaluation protocol, again five-fold cross validation was repeated five times on the dataset to prevent overfitting and reduce any bias due to specific data partitioning. This means that each experimental validation was executed five times. Therein, totally 25 experimental runs were conducted to evaluate the performance of each IDS classifier and the evaluation metrics averaged across the different runs were reported. Also, during each five-fold cross validation, the dataset was randomly shuffled and divided into five sets, out of which one was used as test set while others were used as training set. This experimental evaluation framework is depicted in Fig. 4.

## 7 Experimental results and discussion

This section presents the experimental results that were conducted based on the framework discussed in the previous section 6.3 and analysis of those results to demonstrate the impact of different feature evaluation measures on the efficiency of intrusion detection.

### 7.1 Feature selection analysis

Procedurally, the first experiment was conducted to select the most important features for different classes of attacks applying the four feature evaluation measures discussed in Section 4. Here, the Greedy algorithm was employed as search strategy for the multivariate measures, consistency and correlation to select the most optimal subset of features. While the univariate measures, Information and distance applies a threshold of 25% of the total features, sorted in descending order of importance to select the informative features for each type of attacks.

The features selected by these four feature evaluation measures on NSL-KDD and UNSW-NB15 datasets are reported in Table 3 and Table 4 respectively. Each row lists the indexes of the features selected for each attack class by different feature evaluation measures. The number in parentheses indicates the number of features (NF) selected by the respective feature evaluation measures. These features are represented by feature indexes in table for reasons of brevity. Readers may refer to Table A1 and Table A2 in the Appendix – A for resolving feature index to name.

**Table 3:** Features selected by four feature evaluation measures for different attack classes in NSL-KDD.

Attack class	Correlation		Consistency	
	NF	Feature subset	NF	Feature subset
**DoS**	(6)	{5,6,12,26,30,39}	(5)	{3,5,23,34,39}
**Prob**	(8)	{3,5,6,12,27,29,37,41}	(5)	{3,5,32,35,40}
**R2L**	(3)	{5,10,11}	(4)	{1,3,5,6,37}
**U2R**	(4)	{14,17,18,32}	(7)	{1,5,17}


Attack class	Information		Distance	
	NF	Feature subset	NF	Feature subset
**DoS**	(10)	{3,4,5,6,23,29,30,34,38,39}	(10)	{3,12,26,29,32,33,34,36,38,39}
**Prob**	(10)	{3,5,6,12,33,34,35,36,37,41}	(10)	{2,3,12,31,32,33,34,35,36,40}
**R2L**	(10)	{3,5,6,10,22,23,24,33,36,37}	(10)	{2,3,10,12,22,31,32,33,34,36}
**U2R**	(10)	{1,3,5,10,13,14,17,32,33,36}	(10)	{2,3,12,14,24,31,32,33,34,36}

**Table 4:** Features selected by four feature evaluation measures for different attack classes in UNSW-NB15.

Attack class	Correlation		Consistency	
	NF	Feature subset	NF	Feature subset
**Fuzzers**	(1)	{10}	(11)	{3,7,8,9,10,17,27,28,32,33,35}
**Analysis**	(3)	{2,27,35}	(11)	{2,13,18,27,28,31,34,36,39,40,41}
**Backdoor**	(2)	{2,35}	(6)	{2,3,7,27,28,40}
**DoS**	(2)	{2,10}	(7)	{3,7,8,16,31,40,41}
**Exploits**	(3)	{2,10,32}	(12)	{2,7,8,9,10,17,27,28,31,36,40,41}
**Generic**	(4)	{3,4,11,35}	(8)	{3,7,8,25,27,31,36,40}
**reconnaissance**	(2)	{7,10}	(8)	{2,3,7,12,31,36,40,41}
**Shellcode**	(2)	{10,41}	(8)	{3,7,8,27,31,33,36,40}
**Worms**	(2)	{7,30}	(5)	{3,7,10,15,27}


Attack class	Information		Distance	
	NF	Feature subset	NF	Feature subset
**Fuzzers**	(11)	{7,10,8,32,11,27,13,9,28,17,1}	(11)	{10,11,16,32,42,28,13,20,2,3,40}
**Analysis**	(11)	{7,27,12,2,8,9,28,1,13,32,6}	(11)	{2,3,4,10,16,20,21,22,27,29,42}
**Backdoor**	(11)	{7,2,27,12,8,9,1,28,11,32,10}	(11)	{2,4,10,16,20,27,31,35,36,41,42}
**DoS**	(11)	{7,27,2,12,8,9,1,28,11,32,10}	(11)	{2,10,11,16,20,27,31,32,36,41,42}
**Exploits**	(11)	{7,8,10,27,11,28,32,12,9,1,17}	(11)	{2,11,16,27,28,31,32,35,36,41,42}
**Generic**	(11)	{7,12,35,27,9,1,34,36,32,41,4}	(11)	{3,9,10,16,27,33,34,35,40,41,42}
**Reconnaissance**	(11)	{7,27,12,8,28,10,9,6,32,13,1}	(11)	{2,4,10,16,20,27,31,34,36,41,42}
**Shellcode**	(11)	{7,12,27,10,8,28,9,1,32,6,11}	(11)	{2,3,4,10,16,20,27,29,32,35,42}
**Worms**	(11)	{7,27,28,8,6,15,3,10,25,32,11}	(11)	{2,3,4,10,16,21,22,27,29,32,42}

From the results in Table 3 and Table 4, it can be observed that though there are some overlapping features among the four feature evaluation measures, feature subsets are distinct for different classes of attacks. Also it can be clearly noted that when compared to univariate measures, multivariate evaluation measures such as correlation and consistency are more efficient in eliminating irrelevant/redundant features and selecting the most compact subset of features across all types of attack classes. Within multivariate measures, correlation seems to retain the smallest number of features. Further, in conformity with the time complexity of its Algorithm 2, it was reasonably faster than others despite of its searching strategy. Thus the results in the Table 3 and Table 4, prove the ability of correlation measure in selecting the lowest proportion of features. In general, all the four feature evaluation measures have shown dimensionality reduction by selecting a small proportion of the original features.

### 7.2 Classification accuracy analysis

Generally, all classifiers are not able to take the advantage of all informative features. In this direction, second set of experiments were conducted to investigate the impact of the four feature evaluation measures on detection accuracy of IDS applying four different classifiers. As discussed in previous section, KNN, RF, SVM and DBN were utilized in this context. These four classifiers approach differently the problem of supervised machine learning. Nevertheless, tuned parameter is crucial for improving classifier performance. Therein, the best parameter values for KNN and SVM were selected performing grid search on NSL-KDD and UNSW-NB15. The parameter range used and the reported results of grid search are illustrated in Table 5. Whereas for RF, the default parameters were utilized. Considering the computational complexity, grid search was not used in case of DBN rather the parameter values given in Table 6 that proved to achieve best performance in previous experiment settings were utilized.

**Table 5:** Parameter settings for classifiers.

Classifiers	Parameters	Value range	Optimal value	
			NSL-KDD	UNSW-NB15
SVM	Kernel	[RBF, linear]	linear	linear
	C	[1,10,100,1000]	1000	10
KNN	k	[3,5,10,15]	5	15

**Table 6:** DBN structure.

Parameters	Values
hidden layer structure	[41,41]
activation function	‘relu’
learning rate	0.1
drop out rate	0.2
Pre-training iteration	10
Fine-tuning iteration	100

Intrusion detection accuracy obtained under optimal parameter of each classifier is used here for comparison. The accuracy obtained for four classifiers with four feature evaluation measures over the baseline performance of these classifiers with all features on NSL-KDD and UNSW-NB15 is presented in Table 7 and Table 8 respectively. From these results, it can be observed indeed the effectiveness of applying feature selection not only improves the detection accuracy of minority attack class types but also helps to reduce the data acquisition cost in future minimizing the number of features required to achieve competitive detection accuracy with high dimensional imbalanced network traffic. This clearly confirms our initial discussion that feature selection eliminating irrelevant features can serve as an effective alternative approach to manage the class imbalance problem on high dimensional intrusion datasets.

**Table 7:** Classification Accuracy Analysis of four feature evaluation measures on NSL-KDD.

Attack class	ALL features				Correlation				Consistency			
	KNN	RF	SVM	DBN	KNN	RF	SVM	DBN	KNN	RF	SVM	DBN
**DoS**	99.88	99.96	98.2	99.44	97.7	97.94	96.0	98.4	99.8	99.96	97.5	98.3
**Prob**	99.63	99.84	97.8	98.4	99.67	99.78	97.3	96.9	99.47	99.91	96.8	97.27
**R2L**	99.77	99.90	98.46	98.46	99.01	99.01	98.46	98.4	99.90	99.94	98.46	98.46
**U2R**	99.9	99.93	98.94	98.4	99.93	99.92	99.8	99.85	99.93	99.94	99.9	99.9


Attack class	Information				Distance			
	KNN	RF	SVM	DBN	KNN	RF	SVM	DBN
**DoS**	99.91	99.97	98.4	99.5	98.9	99.0	93.7	92.0
**Prob**	99.69	99.90	98.8	98.8	99.79	99.80	98.7	98.7
**R2L**	99.72	99.91	98.9	98.46	99.73	99.84	98.7	98.84
**U2R**	99.93	99.96	99.9	99.85	99.93	99.93	99.9	99.85

**Table 8:** Classification Accuracy Analysis of four feature evaluation measures on UNSW-NB15.

Attack class	ALL features				Correlation				Consistency			
	KNN	RF	SVM	DBN	KNN	RF	SVM	DBN	KNN	RF	SVM	DBN
**Fuzzers**	80.01	83.52	79.25	79.21	84.7	84.7	75.4	78.4	88.5	90.1	79.2	83.7
**Analysis**	98.97	99.0	96.78	96.78	99.2	99.1	98.8	98.8	99.3	99.4	98.8	98.8
**Backdoors**	99.39	99.4	97.29	97.21	99.4	99.4	99.3	97.6	99.8	99.9	98.8	98.6
**DoS**	96.0	96.61	91.65	91.45	96.1	96.2	91.5	92.3	98.4	99.1	92.2	94.8
**Exploits**	80.36	92.92	73.95	76.4	89.9	91.0	70.8	85.1	97.0	98.8	84.4	85.2
**Generic**	99.37	99.42	95.98	95.97	99.2	99.2	99.0	90.2	99.6	99.7	99.1	99.1
**Reconnaissance**	90.62	92.21	86.87	86.88	97.9	98.1	90.2	89.6	97.8	99.5	86.4	85.7
**Shellcode**	98.56	98.46	97.99	97.99	98.3	98.2	98.0	97.9	99.3	99.67	98.4	97.9
**Worms**	99.73	99.77	99.74	99.74	99.8	99.89	99.7	99.7	99.9	99.99	99.7	99.7


Attack class	Information				Distance			
	KNN	RF	SVM	DBN	KNN	RF	SVM	DBN
**Fuzzers**	87.2	89.07	81.8	83.9	87.3	89.62	83.0	83.9
**Analysis**	98.9	99.33	97.9	97.6	99.0	99.35	99.0	98.8
**Backdoors**	99.5	99.84	97.1	97.9	99.7	99.76	97.1	99.2
**DoS**	97.7	98.85	93.0	91.2	98.5	99.0	95.1	96.8
**Exploits**	95.3	97.56	79.1	84.4	97.6	98.59	86.9	93.0
**Generic**	99.3	99.69	93.2	89.0	99.5	99.71	94.6	96.9
**Reconnaissance**	98.6	99.11	91.9	88.5	98.9	99.54	96.5	97.1
**Shellcode**	98.7	98.63	98.0	97.9	98.7	98.44	98.0	97.9
**Worms**	99.9	99.96	99.7	99.7	99.8	99.90	99.7	99.7

Likewise observing the experimental results of four classifiers using the four feature evaluation measures, it can be seen that RF classifier achieves better detection accuracy compared to its counterparts with all feature evaluation measures across all attack types. Here particularly, it exhibits comparably better with consistency and distance measures in improving the detection accuracy of minority attack class types. The reason possibly might be due to the non-linear “if-then-else” rules underlying the decision tree. These non-linear rules are further enhanced by the feature subset identified by consistency and distance measure to show more accurate detection in a complex detection environment.

Whereas the classifiers KNN, SVM and DBN perform better with consistency, information and distance measures for most of the attack types. Most specifically, KNN classifier achieves better detection accuracy with consistency measure and second better accuracy with distance measure. Hence KNN classifier with consistency measure might be better choice to get benefited with better detection accuracy. Also, it can be noticed that the distance measure being most closely related to the KNN classifier produces better accuracy results with KNN than with other classifiers. Similarly, it can be seen that SVM and DBN classifiers perform better with distance measures for all attack classes except for two attacks, probe and generic.

Another most noteworthy result is that the classifier DBN exhibits comparable performance improvement in detection accuracy with feature selection over the baseline classifiers. Thus demonstrating the effectiveness of feature selection in enhancing its discriminative ability in handling the class imbalance problem on high-dimensional network traffic by avoiding overfitting and reducing the training complexity.

**Figure 5:**
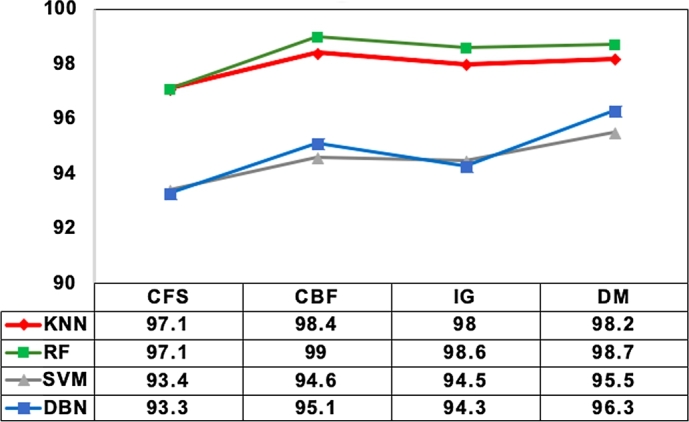
Mean Plot of four classifier groups against four feature evaluation measures.

To verify and ensure the above discussed findings, statistical analysis was conducted computing the mean accuracy value for each classifier against the four feature evaluation measures. The mean plot of four classifier groups comparing the accuracy to four feature evaluation measures is shown in Fig. 5. This plot confirms our findings and gives better understanding of how the mean accuracy varies across the four groups of classifiers.

Overall, it can be summarized that RF is better choice than its counterparts across all attack types especially in detecting minority attack class types. Also, the performance of all classifiers were varied with different feature evaluation measures across attack types. And all the classifiers performed comparably well with only consistency measure for most of the attack classes. This indicates that the consistency measure is more generalizable in identifying the most informative features that minimizes the overlap degree across different attack types and are capable of detecting new attacks.

### 7.3 Performance analysis

As third step of analysis, experiments were conducted to investigate the effectiveness of the four feature evaluation measures on the detection competence of IDS model for different attack classes. For these experiments, four IDS models were built for each attack class using the feature subsets selected by the four feature evaluation measures along the baseline model with all features. Here, RF was used as IDS classifier based on its better performance in the previous experiments. The experimental results on NSL-KDD and UNSW-NB15 datasets with regard to DR, FAR and KS are tabulated in Table 9 and Table 10 respectively.

**Table 9:** Performance Analysis of four feature evaluation measures on NSL-KDD.

Attack class	ALL features			Correlation			Consistency			Information			Distance		
	DR	FAR	KS	DR	FAR	KS	DR	FAR	KS	DR	FAR	KS	DR	FAR	KS
**DoS**	1	0.000	0.999	0.981	0.007	0.998	1.000	0.001	0.999	1.000	0.001	0.999	0.994	0.014	0.98
**Prob**	1	0.004	0.997	0.999	0.039	0.991	1.000	0.004	0.996	1.000	0.005	0.996	0.999	0.009	0.992
**R2L**	1	0.045	0.974	0.998	0.106	0.879	1.000	0.030	0.983	1.000	0.044	0.971	0.999	0.098	0.911
**U2R**	1	0.442	0.69	1.000	0.673	0.404	1.000	0.428	0.704	1.000	0.381	0.739	0.978	0.436	0.691

**Table 10:** Performance Analysis of four feature evaluation measures on UNSW-NB15.

Attack class	ALL features			Correlation			Consistency			Information			Distance		
	DR	FAR	KS	DR	FAR	KS	DR	FAR	KS	DR	FAR	KS	DR	FAR	KS
**Fuzzers**	0.937	0.145	0.780	0.799	0.267	0.659	0.941	0.143	0.784	0.909	0.196	0.710	0.860	0.176	0.653
**Analysis**	0.999	0.116	0.926	0.998	0.186	0.870	0.999	0.11	0.931	0.997	0.125	0.897	0.996	0.142	0.879
**Backdoor**	1	0.037	0.976	1.000	0.183	0.895	1	0.019	0.982	0.999	0.042	0.963	0.999	0.091	0.943
**DoS**	0.997	0.018	0.979	1.000	0.211	0.860	0.996	0.02	0.971	0.998	0.011	0.982	0.993	0.129	0.897
**Exploits**	0.991	0.012	0.978	0.979	0.282	0.801	0.990	0.015	0.975	0.982	0.034	0.947	0.972	0.092	0.925
**Generic**	0.999	0.004	0.996	0.999	0.083	0.983	0.999	0.004	0.995	0.999	0.007	0.990	0.998	0.027	0.987
**Reconnaissance**	0.999	0.006	0.992	0.988	0.126	0.928	0.997	0.014	0.981	0.995	0.029	0.965	0.995	0.084	0.967
**Shellcode**	0.998	0.125	0.893	0.986	0.224	0.619	0.999	0.090	0.916	0.995	0.425	0.618	0.995	0.473	0.582
**Worms**	1	0.208	0.861	1.000	0.292	0.753	1.000	0.062	0.981	1.000	0.069	0.920	1.000	0.123	0.904

From the evaluation metrics of NSL-KDD datasets, it can be clearly observed that all the four feature evaluation measures show satisfactory DR over the baseline for all classes of attacks. But with more precise observation, it is obvious that the IDS models built using the feature subset of multivariate consistency measure and univariate Information measure produced comparably better detection rates for all attack classes than those built using the feature subset of other measures. Also, they demonstrate the best result in FAR and KS. On other hand, observing the performance of correlation and distance measure, it is clear that correlation measure was superior in selecting less number of features but its influence in improving the DR of IDS model maintaining low FAR was not remarkable. Whereas with distance measure, it is found to achieve the third best among the four evaluation measure in influencing the detection capability of IDS model across all attack types. Further, it can be noticed that though, consistency measure proved higher priority than its counterparts in terms of DR, FAR and KS, its achievement of 0.1% FAR with DoS raises slightly to 0.4% in Probe and 3% in R2L classes and further to 55% in U2R. This is might be due to smaller numbers of samples for ‘U2R’, ‘R2L’ and Probe in training set than for DoS classes. Nevertheless, the consistency measure proved to perform best and more stable in identifying the most informative feature subset for all attacks in NSL-KDD datasets.

The experimental results on UNSW-NB15 dataset also conformed that the consistency measure was stable across all attack classes in attaining the highest detection performance in terms of DR and KS. Followed by information measure and distance measure. This is really appreciable. Also FAR achieved by consistency measure across all attack classes were very less which is actually a good requirement for an IDS. Because increase in FAR increases overheads, time and resources of the systems. Overall, consistency-based measure produces most of the best results and has proven their superiority in increasing DR for all attacks especially even with less frequent attack classes like U2R and Worms. It is evident from all the above results that the consistency measure is effective to handle class imbalance problem on high dimensionality problem in IDS.

### 7.4 Statistical analysis

Finally, statistical analysis is carried out as stated in literature [50], [51] to confirm the feature evaluation measure that holds significant difference in contributing to IDS performance. ANOVA is one of the most popular and appropriate hypothesis testing that looks for statistical difference between the output of more than two algorithms and confirms whether the average difference between the outputs is significant or it is due to random chance. Hence it is adopted here for statistical analysis and for answering the NULL hypothesis given below,

**Figure:**
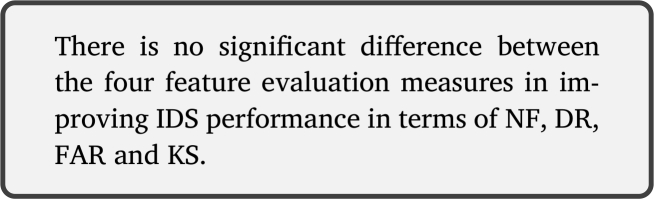

In this respect, ANOVA analysis was conducted on the performance results given in Table 9 and Table 10. As first step, ANOVA statistics was computed on NF, DR, FAR and KS results obtained for both NSL-KDD and UNSW-NB15 datasets, and the findings are presented in Table 11. Observing the results in Table 11, it can be noted that minimum and maximum and 95% confidence interval for mean values of DR and KS results obtained using consistency measure are higher compared to other measures. Also its FAR values are lower compared to other measures. Therefore, it can be stated that the consistency measure provides a better IDS performance among all other measures.

**Table 11:** ANOVA Statistics of performance metrics (NF, DR, FAR and KS) for four feature evaluation measures.

Performance metrics (PM)	Feature measures (FM)	N	Mean	Std. deviation	Std. error	95% confidence interval for mean	
						Lower bound	Upper bound
NF	Correlation	13	3.23	1.921	0.532	2.186	4.275
	Consistency	13	7.46	2.569	0.712	6.064	8.858
	Information	13	10.7	0.480	0.133	10.43	10.95
	Distance	13	10.7	0.480	0.133	10.43	10.95

DR	Correlation	13	0.98	0.055	0.015	0.949	1.008
	Consistency	13	0.99	0.016	0.004	0.985	1.002
	Information	13	0.99	0.025	0.007	0.976	1.003
	Distance	13	0.99	0.024	0.006	0.977	1.003

FAR	Correlation	13	0.21	0.167	0.046	0.115	0.296
	Consistency	13	0.07	0.116	0.032	0.009	0.135
	Information	13	0.11	0.140	0.039	0.033	0.186
	Distance	13	0.14	0.145	0.040	0.066	0.224

KS	Correlation	13	0.82	0.172	0.048	0.724	0.912
	Consistency	13	0.93	0.108	0.030	0.868	0.987
	Information	13	0.89	0.125	0.035	0.830	0.965
	Distance	13	0.87	0.135	0.038	0.976	0.944

The ANOVA statistics encouraged to perform two-way ANOVA analysis between the four feature evaluation measures (FM) and the four performance evaluation metrics (PM) and the findings are presented in Table 9 and Table 10. This analysis was carried out for significance level of 5%, i.e. for 95% confidence level. It can be observed from the results in Table 12 that the interaction between the explanatory variables FM and PM is significant (F=59.56 and sig<0.05). Hence, it is evident to conform that the defined NULL hypothesis remains rejected and the effects of the feature evaluation measure on the outcome of the four performance evaluation metrics differed significantly.

**Table 12:** ANOVA Significance report of feature evaluation measures.

Sources	Sum. Sq.	Diff.	Mean Sq.	F	Sig.
FM	121.8	3	40.60	59.5	3.0e-27
PM	2130.6	3	710.19	1040.91	1.8e-118
FM * PM	366.33	9	40.7	59.66	6.7e-51
Error	131	192	0.68		
Total	2749.72	207			

**Figure 6:**
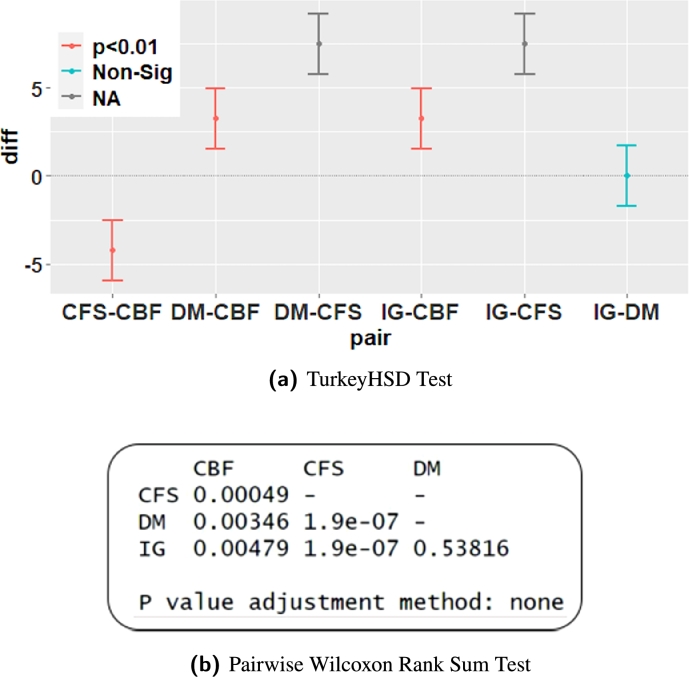
Post hoc ANOVA Analysis for four feature evaluation measures.

**Figure 7:**
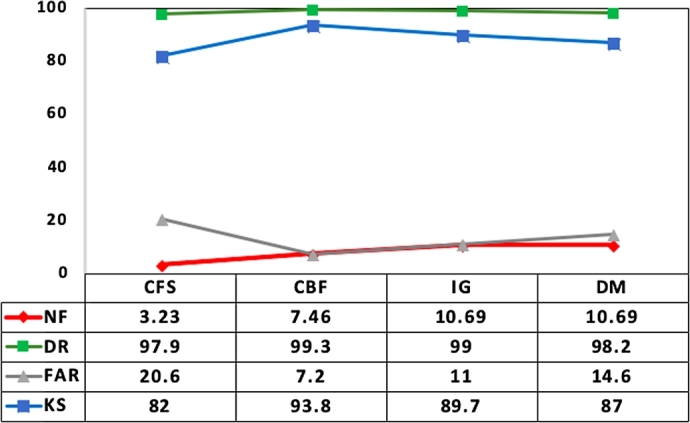
Mean Plot of IDS Performance for four feature evaluation measures.

Accordingly, as third step Post hoc test such as TurkeyHSD and pairwise Wilcoxon rank sum test was carried out to confirm the feature evaluation measure pair that performs significantly different. The results of Post hoc test are given in Fig. 6. It is clear from these results that only the pairs with consistency measure are statistically significant (p<0.05) in improving the detection performance. Added to this, the mean plot for IDS performance in terms of NF, DR, FAR and KS against four feature evaluation measures is shown in Fig. 7 to demonstrate the superiority of consistency measure over its counter part.

From the results of all our experiments, we conclude and recommend the researcher community involved in building IDS to adopt consistency evaluation measure to enhance the classifier performance for intrusion detection.

## 8 Conclusion

This paper presented a comprehensive analysis of four feature evaluation measures namely correlation, consistency, information and distance for intrusion detection. The main goal of the present work was to recommend the best feature evaluation measure that can improve the overall detection performance of an IDS. In this direction, a general experimental framework was designed on two benchmark datasets namely, NSL-KDD and UNSW-NB15 with four different state-of-art machine learning classifiers namely, KNN, RF, SVM and DBN to investigate the influence of the four feature evaluation measures on classification accuracy of an IDS. Under optimized parameter settings, all classifiers provided competitive results; with RF giving the better detection accuracy with all feature evaluation measures. On other hand, all the other classifiers gave the best detection accuracy with consistency measure for most of the attack classes. Further, the effectiveness of the four feature evaluation measures on IDS detection performance in terms of DR, FAR and KS was analyzed. Here all the four feature evaluation measures showed good detection rate for most classes of attacks except for less frequent attack classes like U2R and worms. Only consistency measure was observed to stand out with higher detection rate even for U2R and worms. Also, it surpassed the other measures in achieving low FAR. Thus, consistency measure demonstrated its superiority over others contributing the more critical features for intrusion detection to achieve higher accuracy and detection rate with low false alarm rate. To conclusively conform the most significant feature evaluation measure for intrusion detection, two-step statistical test was conducted. The consistency measure achieved impressive results demonstrating statistically its significance in improving the IDS performance. This versatility of consistency measure demands to recommend it as an appropriate feature evaluation measure for IDS. Taken altogether, the findings from comprehensive analysis are expected to help guide the cybersecurity researchers in designing an effective lightweight system with reduced set of features for the emerging technologies such as IoT and Fog Clouds.

## Declarations

### Author contribution statement

A. Binbusayyis: Conceived and designed the experiments; Analyzed and interpreted the data; Contributed reagents, materials, analysis tools or data; Wrote the paper.

T. Vaiyapuri: Performed the experiments; Analyzed and interpreted the data; Contributed reagents, materials, analysis tools or data; Wrote the paper.

### Funding statement

This research did not receive any specific grant from funding agencies in the public, commercial, or not-for-profit sectors.

### Competing interest statement

The authors declare no conflict of interest.

### Additional information

No additional information is available for this paper.

## Figures and Tables

**Table A1:** Feature List of NSL-KDD dataset.

No.	Feature name	No.	Feature name
1	duration	21	is_hot_login
2	protocol_type	22	is_guest_login
3	service	23	count
4	src_bytes	24	serror_rate
5	dst_bytes	25	rerror_rate
6	flag	26	same_srv_rate
7	land	27	diff_srv_rate
8	wrong_fragment	28	srv_count
9	urgent	29	srv_serror_rate
10	hot	30	srv_rerror_rate
11	num_failed_logins	31	srv_diff_host_rate
12	logged_in	32	dst_host_count
13	num_compromised	33	dst_host_srv_count
14	root_shell	34	dst_host_same_srv_rate
15	su_attempted	35	dst_host_diff_srv_rate
16	num_root	36	dst_host_same_src_port_rate
17	num_file_creations	37	dst_host_srv_diff_host_rate
18	num_shells	38	dst_host_serror_rate
19	num_access_files	39	dst_host_srv_serror_rate
20	num_outbond_cmd	40	dst_host_rerror_rate

**Table A2:** Feature List of UNSW-NB15 dataset.

No.	Feature name	No.	Feature name
1	Id	23	dtcpb
2	dur	24	dwin
3	xProt	25	tcprtt
4	xServ	26	synack
5	xState	27	ackdat
6	spkts	28	smean
7	dpkts	29	dmean
8	sbytes	30	trans_depth
9	dbytes	31	resp_body_len
10	rate	32	ct_srv_src
11	sttl	33	ct_state_ttl
12	dttl	34	ct_dst_ltm
13	sload	35	ct_src_dport_ltm
14	dload	36	ct_dst_sport_ltm
15	sloss	37	ct_dst_src_ltm
16	dloss	38	is_ftp_login
17	sinpkt	39	ct_ftp_cmd
18	Dinpkt	40	ct_flw_http_mthd
19	sjit	41	ct_src_ltm
20	djit	42	ct_srv_dst
21	swin	43	is_sm_ips_ports
22	stcpb	44	attack_cat

## References

[1] da Costa K.A., Papa J.P., Lisboa C.O., Munoz R., de Albuquerque V.H.C. (2019). Internet of things: a survey on machine learning-based intrusion detection approaches. Comput. Netw..

[2] The Economic Impact of Cybercrime—No Slowing Down, Executive Summary, McAfee, 2018.

[3] Vaidya R. (2018). Cyber Security Breaches Survey 2018: Statistical Release.

[4] Morgan S. (2017). Cybercrime Report.

[5] Yaqoob I., Hashem I.A.T., Ahmed A., Kazmi S.A., Hong C.S. (2019). Internet of things forensics: recent advances, taxonomy, requirements, and open challenges. Future Gener. Comput. Syst..

[6] Kimani K., Oduol V., Langat K. (2019). Cyber security challenges for iot-based smart grid networks. Int. J. Crit. Infrastruct. Prot..

[7] Shinder D.L., Cross M. (2008). Scene of the Cybercrime.

[8] Annual Cybersecurity Report, Executive Summary, Cisco, 2018.

[9] Gupta S., Gupta A. (2018). Confronting the new-age cyber-criminal.

[10] Escamilla T. (1998). Intrusion Detection: Network Security Beyond the Firewall, vol. 8.

[11] Liao H.-J., Lin C.-H.R., Lin Y.-C., Tung K.-Y. (2013). Intrusion detection system: a comprehensive review. J. Netw. Comput. Appl..

[12] Buczak A.L., Guven E. (2016). A survey of data mining and machine learning methods for cyber security intrusion detection. IEEE Commun. Surv. Tutor..

[13] Jia Y., Wang M., Wang Y. (2018). Network intrusion detection algorithm based on deep neural network. IET Inf. Secur..

[14] Li J., Zhao Z., Li R. (2017). Machine learning-based IDS for software-defined 5G network. IET Netw..

[15] Dey S., Ye Q., Sampalli S. (2019). A machine learning based intrusion detection scheme for data fusion in mobile clouds involving heterogeneous client networks. Inf. Fusion.

[16] Leite A., Girardi R. (2017). A hybrid and learning agent architecture for network intrusion detection. J. Syst. Softw..

[17] Hajisalem V., Babaie S. (2018). A hybrid intrusion detection system based on ABC-AFS algorithm for misuse and anomaly detection. Comput. Netw..

[18] Viegas E., Santin A., Bessani A., Neves N. (2019). Bigflow: real-time and reliable anomaly-based intrusion detection for high-speed networks. Future Gener. Comput. Syst..

[19] Panigrahi R., Borah S. (2019). Dual-stage intrusion detection for class imbalance scenarios. Comput. Fraud Secur..

[20] Karatas G., Demir O., Sahingoz O.K. (2020). Increasing the performance of machine learning-based IDSs on an imbalanced and up-to-date dataset. IEEE Access.

[21] Ring M., Wunderlich S., Scheuring D., Landes D., Hotho A. (2019). A survey of network-based intrusion detection data sets. Comput. Secur..

[22] Zhang Y., Chen X., Guo D., Song M., Teng Y., Wang X. (2019). PCCN: parallel cross convolutional neural network for abnormal network traffic flows detection in multi-class imbalanced network traffic flows. IEEE Access.

[23] Bace R., Mell P. (2001). NIST special publication on intrusion detection systems.

[24] Viegas E., Santin A., Oliveira L., França A., Jasinski R., Pedroni V. (2018). A reliable and energy-efficient classifier combination scheme for intrusion detection in embedded systems. Comput. Secur..

[25] Moustafa N., Hu J., Slay J. (2019). A holistic review of network anomaly detection systems: a comprehensive survey. J. Netw. Comput. Appl..

[26] Mishra P., Varadharajan V., Tupakula U., Pilli E.S. (2018). A detailed investigation and analysis of using machine learning techniques for intrusion detection. IEEE Commun. Surv. Tutor..

[27] Chattopadhyay M., Sen R., Gupta S. (2018). A comprehensive review and meta-analysis on applications of machine learning techniques in intrusion detection. Aust. J. Inf. Syst..

[28] Kumar V., Minz S. (2014). Feature selection: a literature review. Smart Comput. Rev..

[29] Liu H., Setiono R. (1996). A probabilistic approach to feature selection-a filter solution. ICML.

[30] M.A. Hall, Correlation-based feature selection of discrete and numeric class machine learning, 2000.

[31] Quinlan J.R. (1986). Induction of decision trees. Mach. Learn..

[32] Zhao F., Zhao J., Niu X., Luo S., Xin Y. (2018). A filter feature selection algorithm based on mutual information for intrusion detection. Appl. Sci..

[33] Last M., Kandel A., Maimon O. (2001). Information-theoretic algorithm for feature selection. Pattern Recognit. Lett..

[34] Pes B., Dessì N., Angioni M. (2017). Exploiting the ensemble paradigm for stable feature selection: a case study on high-dimensional genomic data. Inf. Fusion.

[35] Kononenko I. (1994). Estimating attributes: analysis and extensions of relief. European Conference on Machine Learning.

[36] Denoeux T. (1995). A k-nearest neighbor classification rule based on Dempster-Shafer theory. IEEE Trans. Syst. Man Cybern..

[37] Breiman L. (2001). Random forests. Mach. Learn..

[38] Hsu C.-W., Chang C.-C., Lin C.-J. (2003). A Practical Guide to Support Vector Classification.

[39] Yang Y., Zheng K., Wu C., Niu X., Yang Y. (2019). Building an effective intrusion detection system using the modified density peak clustering algorithm and deep belief networks. Appl. Sci..

[40] Le T.-T.-H., Kim Y., Kim H. (2019). Network intrusion detection based on novel feature selection model and various recurrent neural networks. Appl. Sci..

[41] Peng B.-S., Xia H., Liu Y.-K., Yang B., Guo D., Zhu S.-M. (2018). Research on intelligent fault diagnosis method for nuclear power plant based on correlation analysis and deep belief network. Prog. Nucl. Energy.

[42] Taherkhani A., Cosma G., McGinnity T.M. (2018). Deep-FS: a feature selection algorithm for Deep Boltzmann Machines. Neurocomputing.

[43] Zhang H., Li Y., Lv Z., Sangaiah A.K., Huang T. (2020). A real-time and ubiquitous network attack detection based on deep belief network and support vector machine. IEEE/CAA J. Autom. Sin..

[44] Velliangiri S., Pandey H.M. (2020). Fuzzy-Taylor-elephant herd optimization inspired Deep Belief Network for DDoS attack detection and comparison with state-of-the-arts algorithms. Future Gener. Comput. Syst..

[45] Cup K. (1999). http://kdd.ics.uci.edu/databases/kddcup99/kddcup99.html.

[46] Tavallaee M., Bagheri E., Lu W., Ghorbani A.A. (2009). A detailed analysis of the KDD CUP 99 data set. 2009 IEEE Symposium on Computational Intelligence for Security and Defense Applications.

[47] Moustafa N., Slay J. (2015). UNSW-NB15: a comprehensive data set for network intrusion detection systems (UNSW-NB15 network data set). 2015 Military Communications and Information Systems Conference.

[48] Manel S., Williams H.C., Ormerod S.J. (2001). Evaluating presence–absence models in ecology: the need to account for prevalence. J. Appl. Ecol..

[49] Carter J.A., Long C.S., Smith B.P., Smith T.L., Donati G.L. (2019). Combining elemental analysis of toenails and machine learning techniques as a non-invasive diagnostic tool for the robust classification of type-2 diabetes. Expert Syst. Appl..

[50] Xiao B., Benbasat I. (2018). An empirical examination of the influence of biased personalized product recommendations on consumers' decision making outcomes. Decis. Support Syst..

[51] Sarstedt M., Mooi E. (2019). Hypothesis testing and anova. A Concise Guide to Market Research.

